# Temporal changes in ewe vaginal microbiota throughout gestation

**DOI:** 10.3389/fmicb.2024.1359678

**Published:** 2024-02-15

**Authors:** Mackenzie S. Cassas, Lucille C. Jonas, Chiron J. Anderson, Stephan Schmitz-Esser, Curtis R. Youngs

**Affiliations:** ^1^Department of Animal Science, Iowa State University, Ames, IA, United States; ^2^Interdepartmental Microbiology Graduate Program, Iowa State University, Ames, IA, United States

**Keywords:** sheep, vaginal microbiota, 16S rRNA gene amplicon sequencing, Illumina MiSeq, pregnancy

## Abstract

**Introduction:**

Numerous factors are known to influence reproductive efficiency in ewes, but few studies have investigated the potential role of vaginal microbiota in sheep reproductive success. The objective of this study was to thoroughly characterize the ewe vaginal microbiota throughout the course of pregnancy.

**Methods:**

Vaginal samples were collected from 31 pregnant Hampshire and Hampshire X Suffolk crossbred ewes on a weekly basis from pre-breeding to pregnancy testing and then biweekly until just after lambing. To characterize the vaginal microbial communities, DNA was extracted and 16S rRNA gene Illumina MiSeq amplicon sequencing was performed.

**Results and Discussion:**

Alpha diversity metrics indicated an increase in species richness, evenness, and overall diversity throughout gestation. Distinct shifts in the bacterial communities were observed during gestation and were segregated into three periods: early gestation, a transitional period and mid/late gestation. During early gestation, *Actinobacillus*, *Histophilus,* and unclassified *Leptotrichiaceae* were found in greater relative abundance. During the transitional period, a population shift occurred characterized by increasing relative abundance of *Streptococcus* and *Staphylococcus*. During mid/late gestation, *Staphylococcus*, *Streptococcus,* and *Ureaplasma* had the greatest relative abundance. These shifts in the microbial population throughout the ewe’s gestation are likely related to hormonal changes triggered by the growing conceptus, specifically increasing blood concentration of progesterone. The transitional period shift in vaginal microbial communities potentially aligns with the placental take-over of progesterone production from the corpus luteum at approximately day 50 after conception (gestational week 7). Understanding the observed variability of the vaginal microbiota throughout pregnancy will allow for future comparison of ewes that did not become pregnant or had abnormal pregnancies, which could lead to the discovery of potential bacterial biomarkers for pregnancy outcome; this understanding could also lead to development of probiotics to improve sheep reproductive success.

## 1 Introduction

Reproductive success drives profitability of commercial sheep operations whose primary goal is the production of lambs for meat. The ability of a ewe to produce a lamb crop and raise the lamb(s) to weaning each year is an economic necessity for sheep production enterprises. There are a multitude of factors that affect reproductive success or failure of sheep such as age of ewe, season of year, breed of ewe, nutritional status of ewe, environment, and disease (Larsen, 2021). However, limited information exists regarding the ewe vaginal microbiota, and particularly how it changes over the course of gestation and how it might influence sheep reproductive efficiency.

Microorganisms are an important part of the vaginal environment. A normal healthy female reproductive tract harbors beneficial bacteria that allow for the regulation of a healthy pH, inhibition of pathogens, and aid in regulating immune cell function (Torcia, 2019). Pathogenic microbes, or alternatively an imbalance of normal bacteria, have the ability to alter the vaginal environment by causing a detrimental shift in the pH, eliciting an inflammatory response, or negatively affecting sperm capacitation and motility (Zhou et al., 2015; Wang et al., 2020; Ravel et al., 2021). Any alteration to the vaginal or reproductive tract environment has the potential to adversely impact pregnancy outcome.

Studies characterizing the bovine vaginal microbiota have shown differences in microbial population composition due to pregnancy outcome and parity of the female (Laguardia-Nascimento et al., 2015; Deng et al., 2019; Messman et al., 2020). Similarly, the vaginal microbiota of gilts and sows in commercial swine herds (Sanglard et al., 2020) was linked to reproductive outcomes (i.e., high or low farrowing rate).

The sheep vaginal microbiota, like that of other livestock species, has been understudied (Ong et al., 2021; Poole et al., 2023). Cultivation studies characterized the dynamics of ewe vaginal microbial communities when intravaginal sponges were used for synchronization of estrus, and researchers found that intravaginal sponges significantly increased the abundance of bacteria in the vagina. However, the sponges also worked to homogenize the populations of bacteria present regardless of the point in the ewe’s estrous cycle when the sponge was inserted (Manes et al., 2010; Gatti et al., 2011; Martinez-Ros et al., 2018; Quereda et al., 2020). Other studies evaluated the vaginal microbiota of sheep using an alternate method – 16S rRNA gene sequencing (Swartz et al., 2014; Serrano et al., 2020; Koester et al., 2021; Greenwood et al., 2022; Reinoso-Peláez et al., 2023). One of those studies (Koester et al., 2021) was conducted in our laboratory and was based on three sampling time points. A shift of the ewe vaginal microbial communities was observed between the pre-breeding period and the post-pregnancy testing and immediate pre-lambing periods.

The aim of this study was to perform an in-depth characterization of the ewe vaginal microbiota during pregnancy using 16S rRNA gene-based Illumina MiSeq sequencing. Specifically, we aimed to: (1) characterize the sheep vaginal microbiota, starting prior to breeding and continuing until pregnancy testing, to assess potential changes that might occur near the time that pregnancy is established; and (2) characterize the sheep vaginal microbiota from pregnancy testing until the end of gestation (final sample collected within six hours of lambing) to assess the relative stability of the microbial population during continuous exposure to the high blood concentrations of progesterone associated with pregnancy.

## 2 Materials and methods

### 2.1 Ethics statement

All animal procedures in this study were conducted after approval by the Iowa State University Institutional Animal Care and Use Committee (protocol no. 21-141).

### 2.2 Experimental animals

A total of 31 sexually mature Hampshire (*n* = 20) and Hampshire x Suffolk crossbred ewes (*n* = 11) from the Iowa State University sheep teaching farm (42.0308° N latitude, 93.6319° W longitude) were utilized for this study. Estrus was synchronized via insertion of a controlled internal drug-releasing device (CIDR) containing 0.3 g progesterone (EAZI-BREED^™^ CIDR^®^, Zoetis, Inc., Kalamazoo, MI, United States). The CIDR was removed after 10–14 days (to distribute synchronized estrus over a period of a few days to facilitate natural mating by rams). At the time of CIDR removal, ewes were segregated into three separate breeding groups located in three distinct pastures according to the farm’s genetic management program. Each ram was fitted with a marking harness, and dates that ewes were mounted were recorded to pinpoint the day of conception and estimate subsequent lambing dates. Rams were removed at the end of the 34-day breeding period (two 17-day estrous cycles), and ewes were subsequently co-mingled in a single pasture.

From August 1^st^ to November 16^th^, 2021, ewes grazed on pastures consisting of brome grass (*Bromus* spp.), Kentucky bluegrass (*Poa pratensis*), and reed canary grass (*Philaris* arundincea) and were supplemented with 0.9 kg cracked corn (*Zea mays indentata*) per head. From November 17^th^, 2021 and continuing until lambing, ewes were maintained in drylot and were transitioned in late gestation from a mid-gestation ration of brome grass hay (*Bromus* spp.) onto a ration consisting of alfalfa hay (*Medicago sativa*) and cracked corn (*Zea mays indentata*), fed in accordance with daily nutrient requirements for the ewes’ stage of production (NRC, 2007).

Pregnancy testing was conducted using real-time B-mode ultrasound to determine pregnancy status (pregnant or non-pregnant) on November 16^th^, 52 days after removal of breeding rams. Only pregnant ewes that did not have reproductive complications and had a complete set of vaginal samples were analyzed for this study.

### 2.3 Collection of samples

Vaginal swabs were collected by a gloved technician who parted the labia and inserted a sterile 17.8-cm histobrush (Puritan^®^ Histobrush^®^ cytology collection device, Puritan Medical Products, Guilford, ME, United States) approximately 8 cm into the vagina. Upon insertion, the histobrush was spun five times against the vaginal wall. The histobrush was carefully removed from the vagina, ensuring no contact was made between the histobrush and any external surfaces to avoid contamination of the sample. The histobrush was immediately placed inside a sterile 15-ml conical tube containing 10 mL of sterile 1X phosphate-buffered saline solution (PBS, Fisher BioReagents, Fair Lawn, NJ, United States). The end of the histobrush opposite of the bristles was cut with a sterilized cutting tool so that the remaining portion of the histobrush was wholly contained within the 15 mL conical centrifuge tube. The tubes were placed within minutes of collection into a −20°C freezer located at the farm. After sample collection for each day was complete, tubes were transported on ice packs to the laboratory (located less than 10 min from the farm), where they were stored at −80°C until DNA extractions were performed.

**Figure 1:**
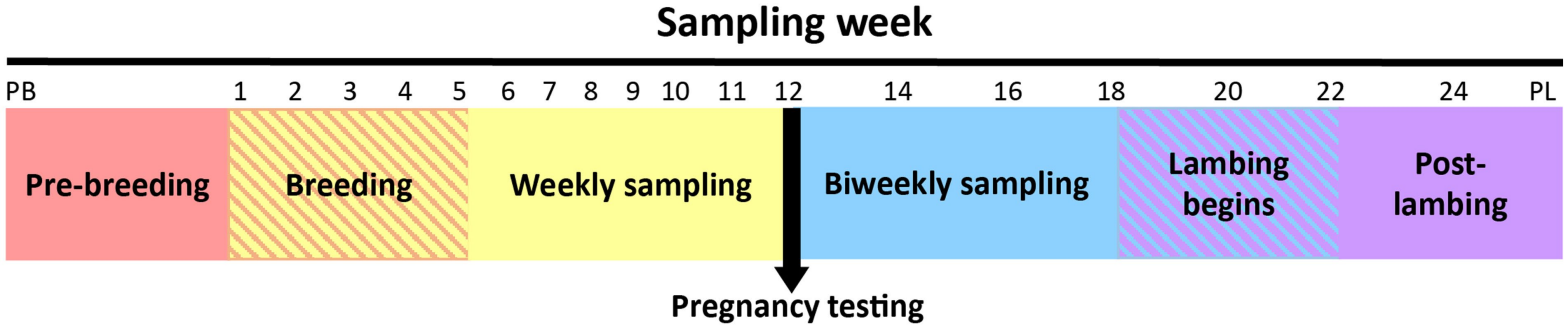
Overview of the ewe vaginal weekly sampling protocol from pre-breeding (PB) until pregnancy testing and bi-weekly sampling protocol from pregnancy testing to post-lambing (PL). Weeks 22 and 24 had fewer samples collected because some ewes had already lambed (crosshatched area). Note that time series is not drawn to scale.

The first sample was collected from all ewes immediately prior to CIDR insertion (designated as the pre-breeding sample). After removal of the CIDR, ewes were introduced to their designated breeding ram. The second sample was taken during the first week of breeding (no samples were collected from ewes while the CIDR was *in situ*). Thereafter, ewes were sampled weekly until pregnancy testing was performed. After pregnancy testing, ewes were sampled biweekly until lambing, which occurred over a 4-week period. A single post-lambing sample was collected within 6 h of parturition. The sampling timeline is depicted in Figure 1.

### 2.4 Sample processing

Samples were processed using a procedure adapted from a previously published protocol (Koester et al., 2021). To extract DNA from the vaginal samples, tubes containing the vaginal swabs were removed from the −80°C freezer and placed into a 37°C water bath until thawed. Samples were removed from the water bath and vortexed vigorously for 10 min to detach sampled material from the histobrush. The brushes were removed, and the tubes were centrifuged at 4°C for 3 min at 4,694 × g (Thermo Scientific Sorvall Legend XR1, ThermoFisher Scientific, Waltham, MA, United States). The supernatant was decanted, and the remaining pellet was re-suspended in 750 μL of the PowerLyzer solution from the DNeasy PowerLyzer PowerSoil kit (Qiagen, Germantown, MD, United States). The DNA was subsequently extracted according to the manufacturer’s instructions supplied with the DNA extraction kit. The Fisherbrand Bead Mill 24 (Fisher Scientific, Fair Lawn, NJ, United States) was used for mechanical cell lysis to physically disrupt cells at a speed of 6 m/s for three one-minute cycles with 10 s rest in between each cycle. The DNA concentrations were determined using a Thermo Scientific NanoDrop 2000 1-position spectrophotometer. Six samples of sterile PCR-grade water (250 μL) were processed with the same DNA extraction kit. These samples were sequenced with the vaginal samples to identify possible contamination during the DNA extraction, amplification, and/or sequencing procedures.

After DNA extraction, samples were diluted with sterile PCR-grade 0.1% diethylpyrocarbonate (DEPC) water (Corning Inc., Corning, NY, United States) to attain a concentration of 50 ng DNA/μl. Samples were sent to the Iowa State University DNA facility for sequencing of DNA using the Illumina MiSeq platform (Illumina, San Diego, CA, United States).

Amplification of DNA was performed using Platinum^™^ Taq DNA Polymerase (Thermo Fisher Scientific, Waltham, MA, United States) with one replicate per sample using universal 16S rRNA gene bacterial primers {515F [5′ -GTGYCAGCMGCCGCGGTAA-3′; (Parada et al., 2016)] and 806R [5′ -GGACTACNVGGGTWTCTAAT-3′; (Apprill et al., 2015)]}, amplifying the hypervariable region V4 as previously described (Kozich et al., 2013). All samples underwent PCR with an initial denaturation step at 94°C for 3 min, followed by 45 s of denaturation at 94°C, 20 s of annealing at 50°C, and extension for 90 s at 72°C. This process was repeated for 35 total cycles; the PCR was finished with a 10-min extension at 72°C. All PCR products were then purified with the QIAquick 96 PCR Purification Kit (Qiagen, Germantown, MD, United States) according to the manufacturer’s instructions. The PCR barcoded amplicons were mixed at equal molar ratios and used for Illumina MiSeq paired-end sequencing with a 250-bp read length and cluster generation with 10% PhiX control DNA.

### 2.5 Sequence analysis

The Mothur V1.48.0 software (Schloss et al., 2009) was used to analyze the sequencing reads using a workflow based closely on the Mothur MiSeq standard operating procedure (Kozich et al., 2013). Version 4.3.1 of the statistical package R (R Core Team, 2023) was used with the Phyloseq package to analyze the OTU abundance data obtained from Mothur (McMurdie and Holmes, 2013). Paired-end reads were merged into contigs in Mothur using the “make.contigs” command. Low quality contigs were removed using the “screen.seqs” command with the following parameters: minimum sequence length threshold of 252 bases, and sequences with ambiguous base calls or homopolymers exceeding eight bases. Chimeric sequences were removed with the “Chimera.vsearch” command using the SILVA Gold database from the Mothur website (Rognes et al., 2016). For the alignment and taxonomic classification of operational taxonomic units (OTUs), the SILVA SSU NR reference database (V138) (Quast et al., 2013) was used. Sequences were clustered into *de novo* OTUs with a cutoff of 99% 16S rRNA sequence similarity (=0.01 distance). To reduce the number of spurious OTUs, all OTUs represented by less than 10 reads were deleted. The decontam R package was used to screen and remove likely contaminant sequences within the data (Davis et al., 2018).

Sixteen samples were removed from the analysis due to insufficient read depth (<5,000 reads), and the remaining samples (*n* = 461) were randomly subsampled to 5,000 reads using R studio with phyloseq to accommodate the sample with the lowest number of reads across the dataset. The subsampled dataset was used for alpha diversity comparison and principal coordinate analysis (PCoA). The full dataset was used for relative abundance graphics and OTU-level comparisons. Once data were screened and the necessary samples were removed, the ggplot R package was used to generate graphics for the sequencing data (Wickham, 2011). Due to the descriptive nature of this study (no treatment groups), no formal statistical analysis was warranted.

### 2.6 Data availability

The 16S rRNA gene sequences were deposited in the NCBI-SRA under Bioproject accession number PRJNA1043118.

## 3 Results

Three ewes were removed from this study because they had abortions. Another ewe was excluded from this study because she died before lambing. Overall, 461 samples from 27 ewes were used in the final analysis (Supplementary Table S1).

### 3.1 Determination of gestation week

Ewes were naturally mated as they exhibited estrus throughout the course of a 34-day breeding period, and, therefore, not all ewes became pregnant on the same day of the breeding season. To assign samples to their corresponding week of gestation, the start of gestation was designated as 147 days before each ewe’s lambing date (the average gestation length of Hampshire sheep). Gestation week ‘0’ refers to the week immediately prior to when gestation began. The staggered lambing dates and bi-weekly sampling schedule resulted in fewer ewes sampled during the second half of gestation.

### 3.2 Overview of the ewe vaginal microbial community

After conducting quality control measures and removing potential contaminant OTUs, a total of 17,490 OTUs remained from the 461 vaginal swabs. The average sequencing depth per sample was 16,550 reads with a standard deviation of 6,611 sequences. The 17,490 OTUs were assigned to 57 phyla, with *Bacillota (*previously known as *Firmicutes), Pseudomonadota (p*reviously known as *Proteobacteria), Actinomycetota,* and *Fusobacteriota* being the four most abundant phyla representing 49.99, 22.43, 10.56, and 6.66% of all reads, respectively (Table 1). These top four phyla represented almost 90% of the sequences. The archaeal phylum *Euryarchaeota* was among the ten most abundant phyla, making up 0.65% relative abundance of reads.

**Table 1:** Relative abundance of the 15 most abundant bacterial and archaeal phyla in all vaginal samples from the ewes in this study.

Phylum	Relative abundance (%)
*Bacillota*	49.99
*Pseudomonadota*	22.43
*Actinomycetota*	10.56
*Fusobacteriota*	6.66
*Bacteroidota*	5.37
*Cyanobacterota*	1.05
*Campylobacterota*	0.79
*Euryarchaeota*	0.65
*Spirochaetota*	0.56
*Planctomycetota*	0.36
*Chloroflexota*	0.28
*Verrucomicrobiota*	0.21
*Bacteria_unclassified*	0.20
*Acidobacteriota*	0.16
*Myxococcota*	0.16

Some notable bacterial families that were present in the vagina of the ewes included *Pasteurellaceae*, *Mycoplasmataceae*, *Staphylococcaceae*, *Leptotrichiaceae*, *Peptostreptococcales*, and *Streptococcaceae* (representing 15.60, 11.32, 8.80, 6.28, 5.38, and 4.79% of all reads, respectively). The most abundant OTU across all weeks was *Ureaplasma* (OTU 2), and it accounted for 10.91% of all reads. OTU 1 (unclassified *Pasteurellaceae*) was the second most abundant OTU making up 8.68% of all the reads, and this OTU was 99.6% similar to *Actinobacillus seminis* when searched with BLAST against the National Center for Biotechnology Information (NCBI) rRNA/ITS database. Other prevalent OTUs were OTU 3 (*Histophilus somni*), OTU 4 (Unclassified *Leptotrichiaceae*), and OTU 6 (*Staphylococcus*), which accounted for 5.04, 3.76, and 3.13% of the reads, respectively. A list of the 50 most abundant sheep vaginal OTUs from pre-breeding to parturition can be found in Supplementary Table S2.

### 3.3 Characterization of sheep vaginal microbiota pre-breeding, throughout gestation, and post-lambing

**Figure 2:**
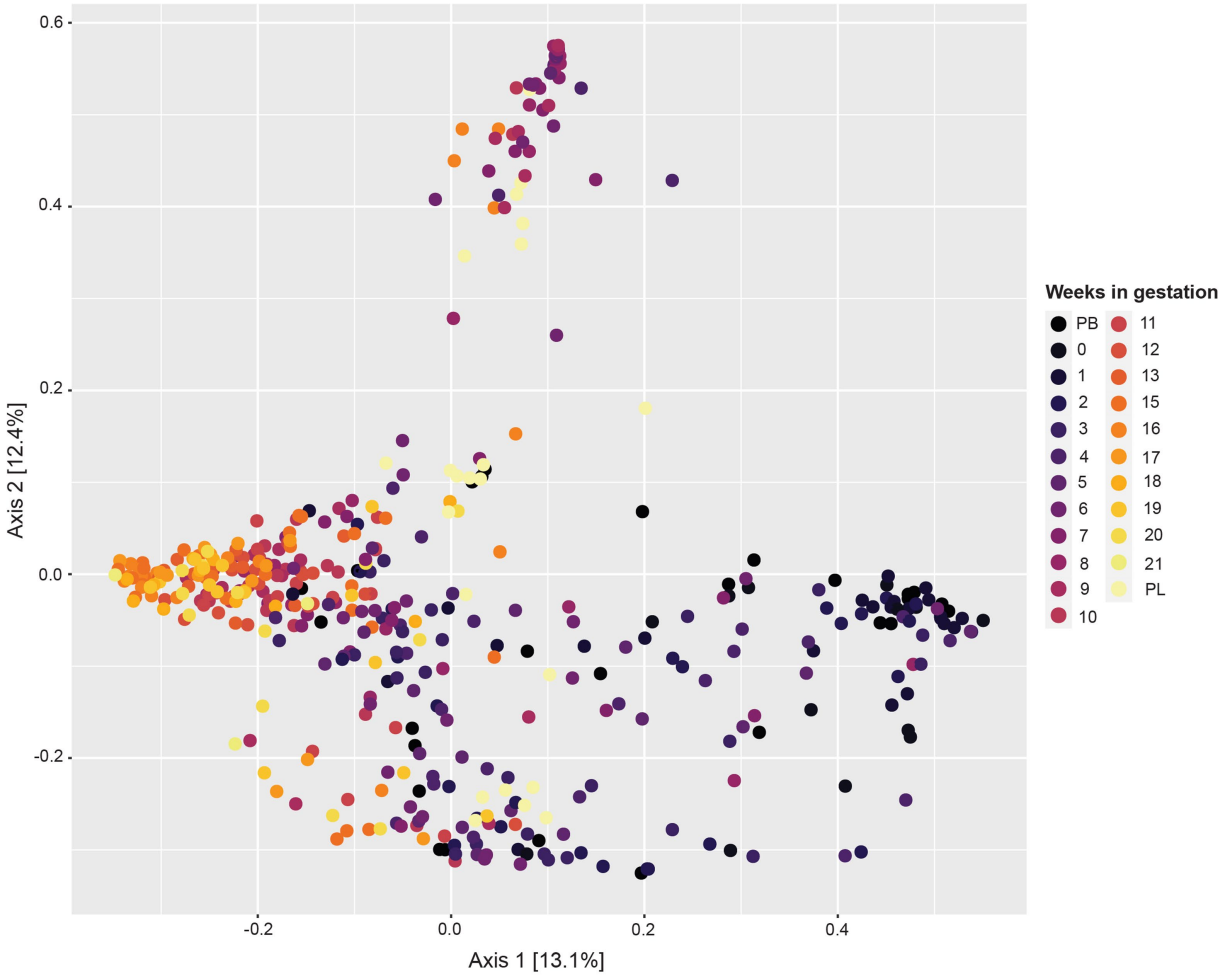
Principal coordinate analysis (PCoA) based on Bray–Curtis dissimilarity measures showing similarity in beta-diversity of sheep vaginal microbial communities across different gestation weeks beginning pre-breeding (PB), continuing throughout gestation and ending post-lambing (PL).

The principal coordinate analysis (PCoA) plot for this data (Figure 2) revealed variability between samples differing by week of gestation. Samples from pre-breeding to the third week of gestation clustered together. Moreover, samples from gestation week 12 to post-lambing clustered distinctly together. Samples from gestation weeks 7 to 10 have more variability, and some cluster in their own grouping as well.

**Figure 3:**
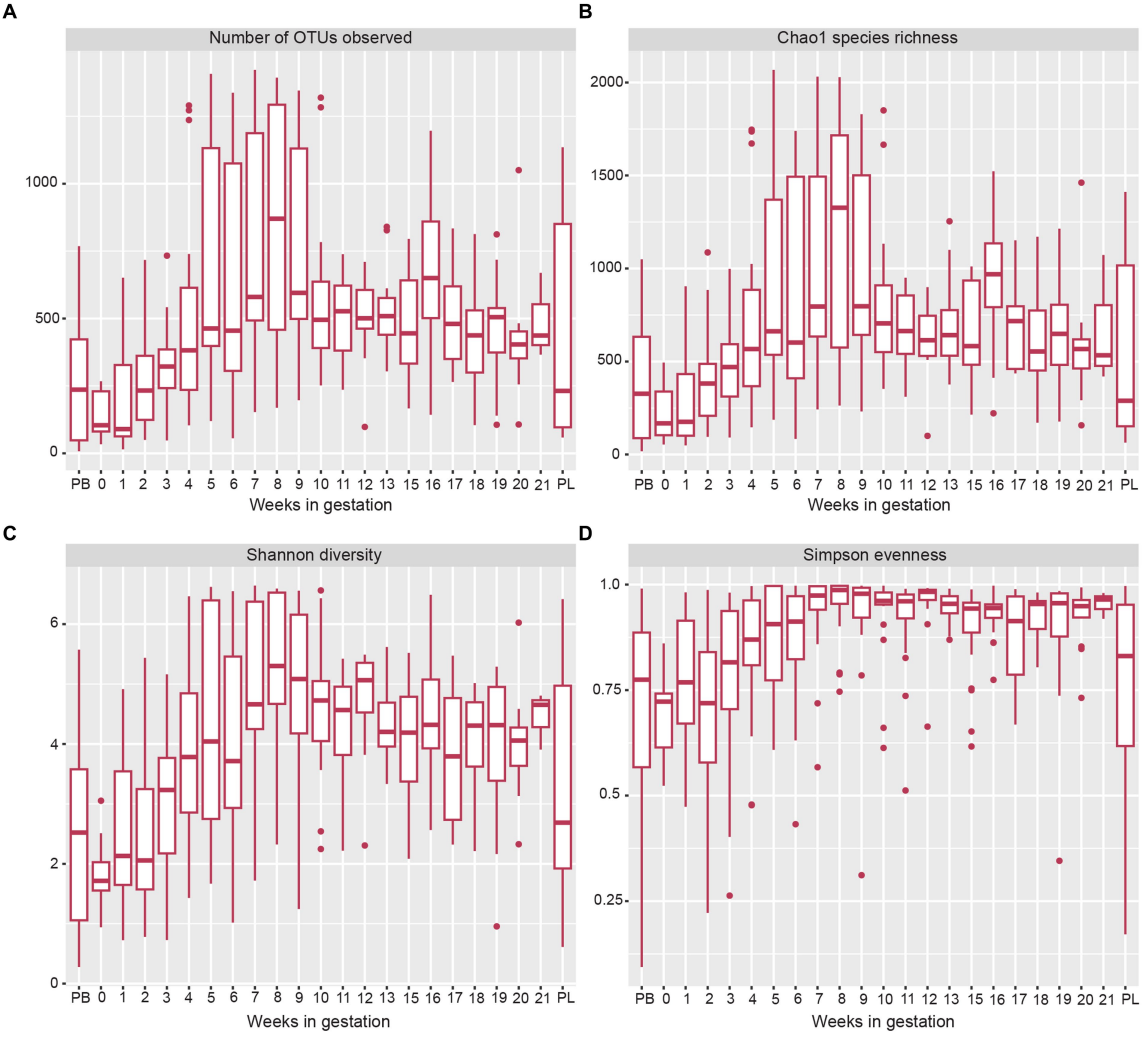
Box and whisker plots of measures of alpha-diversity indices of sheep vaginal microbial communities beginning pre-breeding (PB), continuing throughout gestation and ending post-lambing (PL). **(A)** indicates the number of OTUs observed, **(B)** displays Chao1 species richness, **(C)** shows Shannon species diversity, and **(D)** indicates Simpson species evenness. The box and whisker plot horizontal lines display the lower quartile, median, and upper quartile values of samples in each time point. Dots represent outlier sample values that fell outside of the upper and lower quartiles.

The alpha diversity measures showed several changes in the microbial OTUs present throughout gestation (Figure 3A). The estimated species richness (or Chao) indices showed a steady increase during gestational weeks 1 to 7 and reached their maximum value at week 8 (Figure 3B). From gestation week 9 to post-lambing, species richness values declined closer to levels found in early gestation. When observing overall species diversity (Shannon index; Figure 3C), there was an increase in species diversity from pre-breeding until gestation week 8, after which species diversity decreased somewhat until post-lambing. The Simpson species evenness index (Figure 3D) showed a similar trend with an increase in species evenness until gestation week 8, when it decreased somewhat and stabilized until post-lambing. The immediate post-lambing samples more closely resembled the species evenness of samples taken prior to gestation week 8.

**Figure 4:**
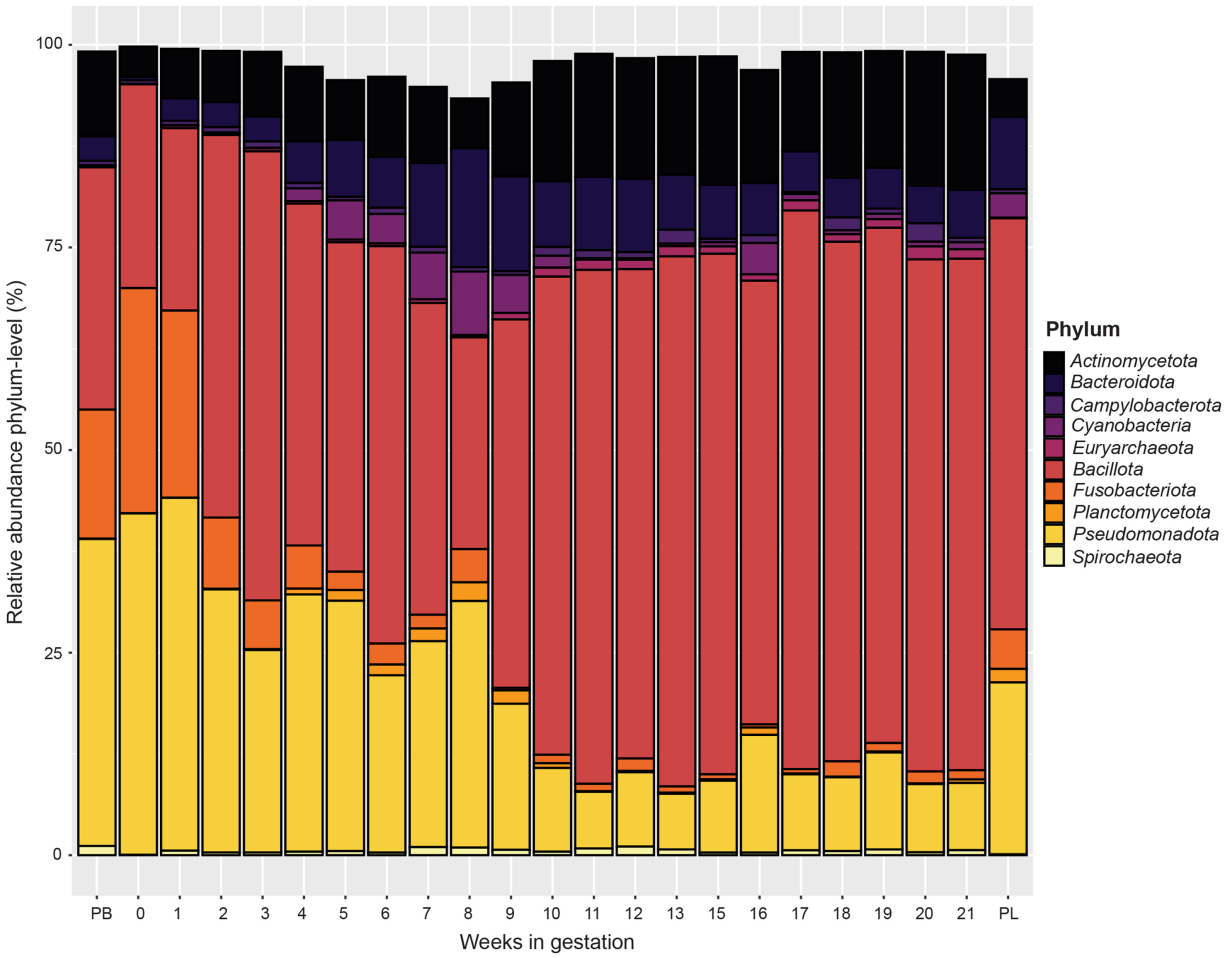
Relative abundance of the 10 most abundant bacterial and archaeal phyla in the ewe vaginal microbiota by pre-breeding (PB), gestation week, and post-lambing (PL) sampling time points. Ewes were sampled every week until pregnancy testing and biweekly thereafter. The final sample (PL) was obtained within 6 h of lambing.

When examining phylum level data (Figure 4), the relative abundance of *Pseudomonadota* and *Fusobacteriota* decreased from pre-breeding to gestational week 9. From gestation week 10 to 21, the relative abundance of *Pseudomonadota* and *Fusobacteriota* plateaued. The relative abundance of *Pseudomonadota* for the post-lambing samples was similar to that of gestation week 9. The phylum *Bacteroidota* increased in relative abundance from gestation week 1 to a peak at week 8. From gestation weeks 9 to 21, *Bacteroidota* relative abundance decreased somewhat as gestation progressed. The phyla *Bacillota* and *Actinomycetota* increased in relative abundance as ewe gestation progressed.

**Figure 5:**
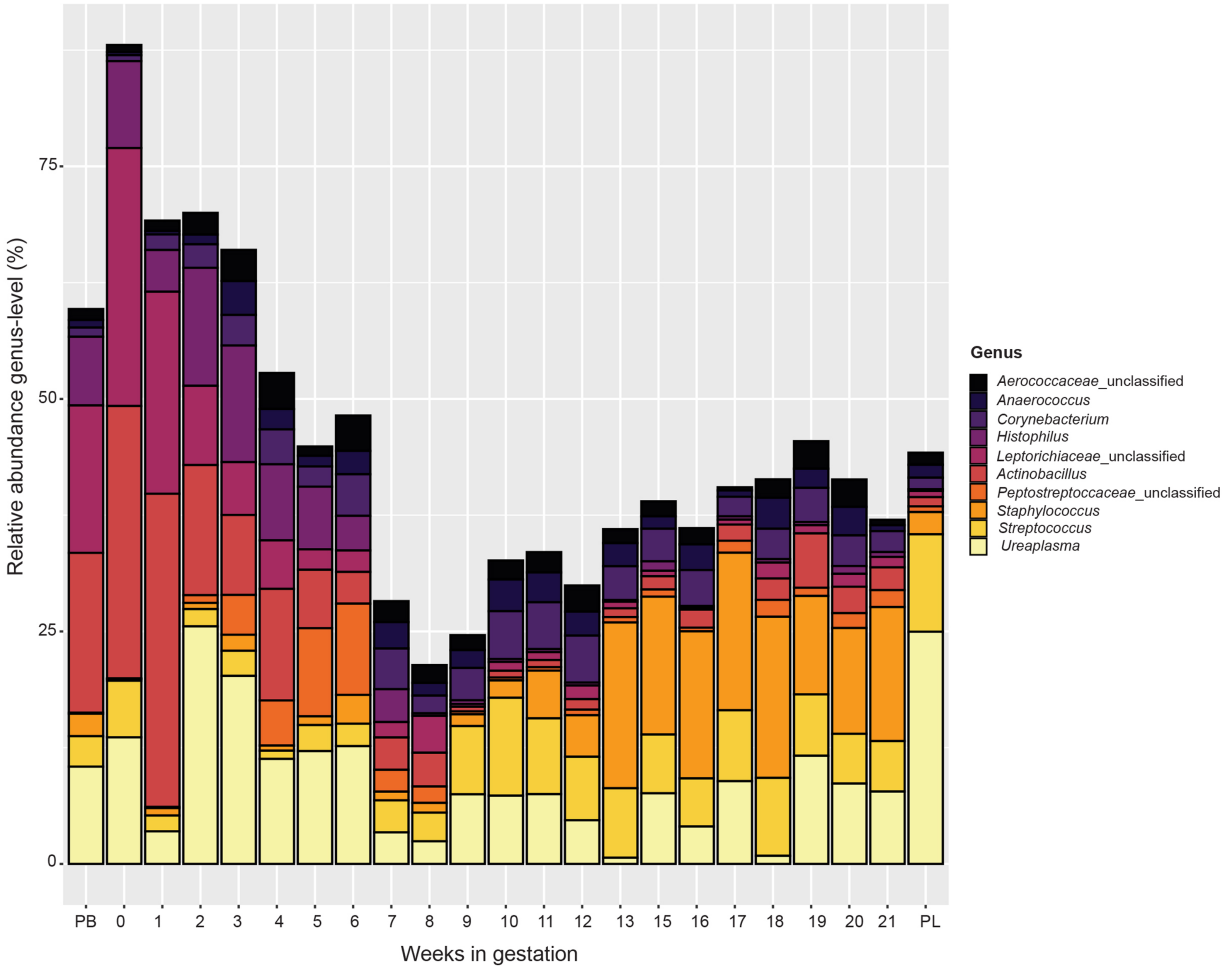
Relative abundance of the 10 most abundant bacterial genera in the vaginal microbiota by pre-breeding (PB), gestation week, and post-lambing (PL) sampling time points. Ewes were sampled every week until pregnancy testing and biweekly thereafter. The final sample (PL) was obtained within 6 h of lambing.

At the genus level (Figure 5), similar to the phylum level data, microbial communities varied by week of gestation. Samples from pre-breeding until gestational week 8 were more similar in their microbial makeup with *Actinobacillus*, unclassified *Leptotrichiaceae, Histophilus*, and *Ureaplasma* composing a greater percentage of the vaginal microbiota. Taxonomic composition for later weeks of gestation (weeks 9 to 21) appeared more similar to each other than to those in early gestation. The overall relative abundance captured by the top 10 bacterial genera decreased from pre-breeding to gestation week 8 and increased slightly for the remainder of gestation to post-lambing. Unclassified *Peptostreptococcaceae* appeared in higher abundance in samples from weeks 3 to 6 of gestation. For gestation week 9 through post-lambing samples, the proportions of *Streptococcus* and *Staphylococcus* were higher than those of earlier weeks. *Corynebacterium* was more abundant in samples from weeks 3 to 20 of gestation. Finally, Unclassified *Aerococcaceace* and *Anaerococcus* were present throughout gestation, although these genera were slightly more abundant during gestational weeks 10 to 21.

## 4 Discussion

This study is the first to characterize the ewe vaginal microbiota at multiple consistent time points throughout gestation. By taking samples at weekly intervals for 12 weeks (until pregnancy testing) and at bi-weekly intervals thereafter until lambing, it was possible to observe the detailed timing of taxonomic modulations of the ewe vaginal microbiota. Microbial populations present in the reproductive tract can affect numerous biological processes, including reproductive function. Specifically, the vaginal microbiota has the potential to impact the reproductive success or failure of a ewe, which in turn influences the profitability of a sheep production enterprise. This study clearly documented shifts in ewe vaginal microbial communities throughout gestation.

The alpha diversity metrics indicated an increase in species richness, evenness, and overall diversity throughout the first 8 weeks of gestation, after which, all measures decreased and stayed more constant until post-lambing. While this increase in the diversity of the vaginal microbiota was consistent with a previous study from our laboratory (Koester et al., 2021), previous studies have not characterized alpha diversity across multiple gestational time points. Whether high microbial diversity is damaging or beneficial depends on the circumstance (Franasiak and Scott, 2015). Because this study only reported data from ewes with positive reproductive outcomes, the increase in the ewe vaginal microbial diversity over the course of gestation presumably is associated with successfully established pregnancies. In addition, more diverse communities may add more resiliency to the vaginal microbiota, allowing the microbes to respond to external factors while avoiding deficits in host health (LeBlanc, 2023).

Based on the observed shifts of the microbial population during the course of this investigation, results can be best described by dividing samples into three distinct gestational time periods: early gestation, a transitional period, and late gestation.

### 4.1 Early gestation

The phyla *Pseudomonadota* and *Fusobacteriota* had greater relative abundance from the pre-breeding time point through gestation week 6 than subsequent weeks. *Actinobacillus* (OTU1) and *Histophilus* (OTU3) made up the largest proportion of reads in the *Pseudomonadota* phylum. *Actinobacillus seminis* causes reproductive disorders in sheep and has been isolated from the genital tract of both ewes and rams (Walker and LeaMaster, 1986). This bacterium has been implicated in ram epididymitis (Baynes and Simmons, 1960) as well as gangrenous mastitis in ewes (Baynes and Simmons, 1960; Watt et al., 1970). Given the high relative abundance of *Actinobacillus* in pregnant ewes, the role of *Actinobacillus* in the ewe reproductive tract warrants further research.

Similar to *Actinobacillus*, species in the genus *Histophilus* are known reproductive pathogens that can result in ewe abortion (Rahaley, 1978; Corbeil, 2007; O’Toole and Sondgeroth, 2016). These bacteria also infect rams and are capable of causing epididymitis, resulting in subsequent infertility (Palomares et al., 2005; Corbeil, 2007). Individual strains of *Histophilus* have the potential to be pathogenic or commensal and can transition between the two classifications depending on environmental conditions (Sandal and Inzana, 2010). In this study, ewe vaginal microbiota community structure was analyzed using Illumina MiSeq 16S rRNA gene amplicon sequencing. The closest related sequence similarities reported here rely on short (253 bp) PCR amplicons. It is widely recognized that classifications based on short 16S rRNA gene amplicons provide only limited taxonomic resolution. Thus, all similarities and closest related species reported in this manuscript represent approximations only. Given that *Histophilus* was highly abundant in this study of ewes with successful pregnancies, it seems reasonable to assume that *Histophilus* acted as a commensal inhabitant of the vaginal microbiomes in this population. However, in a sheep artificial insemination (AI) study, *Histophilus* was more abundant in non-pregnant ewes than pregnant ewes and was significantly more abundant at two farms with higher AI failure (Serrano et al., 2020). This association was verified in another study (Koester et al., 2021) where *Histophilus* was found in greater abundance in non-pregnant ewes compared with ewes that were successfully mated.

A member of the *Fusobacteriota* phylum, unclassified *Leptotrichiaceae*, was present in higher abundance during the beginning of gestation than in later weeks*. Leptotrichiaceae* was considered as normal vaginal microbiota of both Suffolk and Merino ewes during maintenance (Greenwood et al., 2022) as well as in Rambouillet ewes (Swartz et al., 2014). Conversely, *Leptotrichiaceae* was found in greater relative abundance in the vagina of ewes that were not successfully mated when compared to ewes that became pregnant (Koester et al., 2021).

*Bacteroidota* increased in relative abundance throughout early gestation. The genera *Bacteroides, Prevotella,* and *Porphyromonas* were the most abundant members of *Bacteroidota* in this study, representing 0.29, 0.63 and 0.55%, respectively, of the reads collected in this study. There is limited information about the impact of *Bacteroides, Prevotella,* or *Porphyromonas* colonization of the vaginal tract in sheep on reproductive performance. However, all three genera have all been associated with cattle experiencing reproductive maladies such as metritis, endometritis, or vaginitis (Elad et al., 2004; Bicalho et al., 2017; Cunha et al., 2018; Kudo et al., 2021).

The phylum *Bacillota* was also relatively abundant in ewes in this study and increased in abundance from early to late gestation. The genus *Ureaplasma* (represented by OTU2) was the most abundant genus within this phylum. *Ureaplasma* is a common bacterial genus that inhabits the reproductive and urinary tracts of humans and livestock species. In a previous study, *Ureaplasma* was the most abundant OTU of the vaginal microbiota collectively in both pregnant and non-pregnant ewes (Koester et al., 2021). Another recent study showed that *Ureaplasma* was the second most abundant genus in the ewe vaginal microbiota and was also found in ram sperm (Serrano et al., 2020). In most cases, this bacterium is a commensal part of the reproductive microbiota; however, in rare instances, *Ureaplasma* can overgrow leading to inflammation of tissue and resultant vaginitis. *Ureaplasma diversum* contributes to development of granular vulvovaginitis and overall poor reproductive performance in cattle (Pilaszek and Truszczynski, 1988; Diaz et al., 2019).

Unclassified *Peptostreptococcacae* is an additional genus belonging to the *Bacillota* phylum that was most relatively abundant from gestation week 3 to week 6. This bacterium is commensal and known to inhabit the genital, urinary, and oral mucosa of humans (Mekhalif et al., 2022). *Peptostreptococcacae* is also often present in the vaginal microbiota of ewes (Koester et al., 2021). Although not typically pathogenic, it does have the ability to cause vaginosis under certain conditions (Cheng and Wang, 2009).

Given that Peptostreptococcacae and *Ureaplasma* are known bacterial inhabitants of reproductive tracts, it is not surprising that these genera make up a substantial portion of the bacteria present during these early gestational weeks. The *Actinobacillus* and *Histophilus* genera contain species that are known pathogens in sheep, and the presence of these potentially pathogenic bacteria within a healthy and reproductively successful flock demonstrates the need for more investigation into the potential species-specific mechanisms of pathogenicity for these organisms.

### 4.2 Transitional period

Samples taken during gestation weeks 7 to 10 revealed a transitional period of the ewe vaginal microbiota. The number of OTUs observed, species richness, evenness, and overall diversity all reached their highest values at week 8 of gestation. From there, these alpha diversity measurements decreased and stabilized from week 10 until lambing. These trends are clearly depicted in Figure 5 that examines the genus-level community composition throughout ewe gestation. During weeks 7, 8 and 9, the proportion of relative abundance captured by the ten most abundant genera was much lower than that of previous gestational weeks. This observation agrees with the increases in alpha diversity measures as the microbial community structure of the samples during this time period were also more evenly distributed among taxa and diverse in general.

The taxa present in pregnant ewe vaginal microbiomes clearly switch in composition between gestational weeks 8 and 9. Samples from early gestation to gestational week 8 were mostly comprised of unclassified *Leptorichiaceae, Actinobacillus,* unclassified *Peptostreptococcacae, Ureaplasma,* and *Histophilus*. Beginning at gestational week 9 and continuing throughout pregnancy, the composition of the ewe vaginal microbiomes became dominated by *Streptococcus* and *Staphylococcus.* Because this is the first study in which the vaginal microbiome of sheep was observed weekly throughout gestation, there are no other data for comparison of this shift in microbial community structure. The exact physiological cause of this change is unknown but is possibly linked to increased blood concentrations of the steroid hormone progesterone caused by *de novo* progesterone production by the placenta.

### 4.3 Mid/Late gestation

Starting at week 9, the relative abundance of *Pseudomonadota* and *Fusobacteriota* phyla decreased while *Bacillota* and *Actinomycetota* increased. By week 11, the relative abundance of these phyla remained mostly constant through the remainder of gestation. Collectively, these phyla accounted for most of the bacteria present. Within the *Bacillota, Staphylococcus* and *Streptococcus* were most abundant during this period. *Staphylococcus* is commonly found in the ewe vaginal tract (Manes et al., 2010; Swartz et al., 2014). *Staphylococcus*, much like *Streptococcus*, causes mastitis; however, it also has been isolated from various body sites of healthy sheep (Vasileiou et al., 2019). Other members of the *Bacillota* phylum (unclassified *Aerococcaceace* and *Anaerococcus*) were also observed in this study but were more abundant during later gestation. *An*a*erococcus* was present at low numbers in healthy sow and human vaginal microbiotas (Xiao and Liao, 2012; Liang et al., 2022). However, in humans, overgrowth of *Anaerococcus* caused vaginosis or other reproductive tract ailments (Srinivasan and Fredricks, 2008; Kalia et al., 2020). *Aerococcus* was present in the vaginal microbial communities in this study and is associated with bacterial vaginosis in women (Ling et al., 2010). With this said, there are limited data characterizing the effect of either of these genera in the ewe vaginal tract.

### 4.4 Gestational physiology of the ewe in relation to the vaginal microbiota

The shift in the microbial population throughout the ewe’s gestation is most likely related to and perhaps caused by the drastic hormonal changes triggered by the growing conceptus. Various gut and oral bacteria are able to produce, degrade and modify sex steroids such as estrogens (Cornejo Ulloa et al., 2021; Cotton et al., 2023). It also has been shown that in humans, female sex hormones can alter the production of host metabolites in the reproductive tract, such as glycogen (Gregoire and Parakkal, 1972; Miller et al., 2000). Alteration in glycogen levels changed the vaginal microbiota composition (Kaur et al., 2020). Whether bacteria in the reproductive tract have the capacity to modify sex steroids or are responding to changes in host metabolites in relation to hormone modulations is currently unclear and warrants future research.

Sexually mature ewes that have adequate body condition, are in good health, and are not in a period of seasonal anestrus will exhibit an estrous cycle every 17 days until they become pregnant or until they enter into seasonal anestrus. After ovulation (release of an egg from the ovarian follicle), if a ewe is not mated or if the mating is unsuccessful, prostaglandin F2α (PGF) will be released from the endometrium to lyse the progesterone-producing ovarian structure called the corpus luteum (CL), giving the ewe another opportunity to ovulate and become pregnant. In the event that an ovulated egg is fertilized, the embryo will develop and subsequently produce and release ovine trophoblast protein-1 (oTP-1) which prevents PGF from being released and killing the CL, thereby sustaining high blood concentrations of progesterone and facilitating maintenance of pregnancy (Spencer and Bazer, 2004). As the embryo develops, it gives rise to the placenta which not only is responsible for metabolic exchange between the dam and fetus but also acts as an endocrine organ producing, among other compounds, progesterone. Although the CL is the sole source of progesterone during early pregnancy, it is not required throughout the entirety of ewe gestation to maintain pregnancy; the placenta almost completely takes over progesterone production by day 50 of pregnancy (Casida and Warwick, 1945). The 50-day mark during pregnancy in which the placenta takes over progesterone production from the ovary is aligned with the gestation week 7 time point where the observed microbial population shift occurred. More research is needed to ascertain if our hypothesis that the shifts in microbial populations in the early part of sheep pregnancy is caused wholly or in part by conceptus secretions (e.g., oTP-1 and placental-origin progesterone) is correct.

## 5 Conclusion

This study provided an in-depth characterization of the sheep vaginal microbiome over the course of gestation via analysis of samples collected at weekly and then bi-weekly intervals. Results revealed the first detailed insights into the ewe’s vaginal microbiota throughout pregnancy and showed distinct shifts in the microbial population, especially in early pregnancy. Additional studies are needed to fully elucidate the underlying causes and biological relevance of these changes to the ewe vaginal microbiota.

## Data availability statement

The datasets presented in this study can be found in online repositories. The names of the repository/repositories and accession number(s) can be found in the article/Supplementary material.

## Ethics statement

The animal study was approved by the Iowa State University Institutional Animal Care and Use Committee (protocol no. 21-141). The study was conducted in accordance with the local legislation and institutional requirements.

## Author contributions

MC: Data curation, Formal analysis, Investigation, Methodology, Visualization, Writing – original draft, Writing – review & editing. LJ: Data curation, Formal analysis, Investigation, Methodology, Visualization, Writing – original draft, Writing – review & editing. CA: Data curation, Formal analysis, Investigation, Methodology, Visualization, Writing – original draft, Writing – review & editing. SS-E: Conceptualization, Data curation, Investigation, Methodology, Project administration, Resources, Supervision, Writing – original draft, Writing – review & editing. CY: Conceptualization, Data curation, Formal analysis, Funding acquisition, Investigation, Methodology, Project administration, Resources, Supervision, Writing – original draft, Writing – review & editing.
